# Myocardial contractile dispersion: A new marker for the severity of cirrhosis?

**DOI:** 10.15171/jcvtr.2019.25

**Published:** 2019-06-30

**Authors:** Alireza Moaref, Mahmood Zamirian, Hamed Mirzaei, Amin Attar, Elham Nasrollahi, Yaser Bahramvand

**Affiliations:** ^1^Department of Cardiovascular Medicine, Shiraz University of Medical Sciences, Shiraz, Iran; ^2^Cardiovascular Research Center, Shiraz University of Medical Sciences, Shiraz, Iran; ^3^Students’ Research Committee, Shiraz University of Medical Sciences, Shiraz, Iran

**Keywords:** Cirrhotic Cardiomyopathy, QT Dispersion, Tissue Doppler Imaging, Myocardial Contractile Dispersion

## Abstract

***Introduction:*** Cirrhotic cardiomyopathy (CCM) develops in about half of all cirrhotic patients, affecting the long-term morbidity and mortality. Although some studies have shown an increased QT-interval in cirrhotic patients, no evidences of myocardial contractile and QT dispersion (QTd) changes are available. This study aimed to compare myocardial contractile dispersion (MCd), using tissue Doppler imaging (TDI), as well as QTd between cirrhotic patients and healthy individuals, investigating their associations with cirrhosis severity.

***Methods:*** This prospective cross-sectional study was conducted on patients with confirmed liver cirrhosis and healthy individuals. Participants with structural heart disease, heart ventricular pacing, electrolyte abnormalities, using drugs affecting QT interval were excluded. All individuals underwent 2D echocardiography, and TDI by vivid E9 echo machine. MCd and QTd were considered as main outcomes. Chi-square, independent-sample t test, and Pearson correlation test, were used for statistical analyses by SPPS version 17.0. *P * value <0:05 was considered statistically significant.

***Results:*** Sixty participants (40 male/20 female) with a mean age of 40.1 ± 7.1 years in two groups of cirrhotic patients (n=30) and healthy individuals (n=30) were studied. Both groups were statistically similar in terms of age (*P *= 0.31) and gender (*P *= 0.39). MCd and QTd of cirrhotic patients were significantly higher than healthy individuals (MCd: 41.0 ± 26.8 versus 27.6±18.1; *P *= 0.028; and QTd: 37.0 ± 22.1 versus 25.3 ± 8.9; *P *= 0.010). Cirrhotic patients with MELD score <15 had a lower MCd in comparison to score ≥15 (29.2 ± 13.8 versus 50.0 ± 31.1, *P *= 0.034).

***Conclusion:*** Cirrhosis was associated with increased MCd, assessed by TDI. Also, MCd and QTd were associated with a higher MELD score. According to the results, it seems that MCd and QTd might be useful predictor of ventricular arrhythmia and negative prognostic factor in cirrhotic patients.

## Introduction

Cirrhotic cardiomyopathy (CCM) is a term for a group of cardiovascular manifestations develops in about half of cirrhotic patients, affecting markedly on long-term morbidity and mortality.^1^ The most common presentations for CCM are left ventricle (LV) diastolic function impairment, increased baseline cardiac output, as well as abnormal cardiac serologic markers in the absence of cardiac diseases.^2,3^ Autonomic nervous system (ANS) is another system which its changes have been believed to occur during cirrhosis.^3,4^ Recent studies have shown the evidences of cardiac autonomic neuropathy (CAN) through different parameters including prolongation of QT interval (QTi), variability of heart rate (HRV) and blood pressure changes in patients with CCM.^4,5^

QTi prolongation is considered as a probable risk factor for a higher ratio of sudden death and cardiac mortality.^6^ Moreover, some other studies believe that prolonged QTi is related with a greater severity of cirrhosis.^7^ QT dispersion (QTd), maximum minus minimum QTi, has been defined as the interlead corrected QT (QTc) variability, and as a simple and estimated measure of a repolarization general abnormality.^8^ Increased QTd is a direct reflection of disparities in myocardial recovery; thus determination of QTd, as a noninvasive and inexpensive technique, may help to predict arrhythmic events in cirrhotic patients.^4,8^ However, most reports in the literature regarding the effects of cirrhosis focused more on the QTi and corrected QTc and there few study which focused on other parameters likes QTd.^4,8,9^

Furthermore, QTc prolongation is a sign of action potential duration prolong which is considered to be associate with a prolonged LV contraction.^4,9^ However, the ability of electrocardiography (ECG) is known limited to detect regional differences in LV electrical pattern.^4,7,8^ Tissue Doppler imaging (TDI), is known as a clinical method for the quantification of regional myocardial function and the evaluation of (MCd), as an index for detailed time intervals in different regions of the LV.^10^ Besides, to the best of our knowledge, to date there has not been any study to evaluated the association of MCd, using TDI, with cirrhosis and its severity. This study was aimed to evaluate MCd tissue study and QTd in cirrhotic patients, investigating their associations with cirrhosis severity.

## Materials and Methods

### Study design and setting

This prospective cross-sectional study was conducted to on cirrhotic patients and healthy individuals referred to Department of Organ Transplant, Nemazi Hospital, Shiraz, Iran between 2016/July and 2017/May. Patients with a baseline ECG abnormality such as bundle branch block, Wolf-Parkinson-White, left ventricular hypertrophy, and other pathological ECG conditions were excluded from study.

### Participants

Patients with confirmed liver cirrhosis diagnosis were enrolled. Cirrhosis was diagnosed based on the generally accepted criteria: history, physical examinations, laboratory tests, imaging examinations (i.e. panendoscopy), abdominal ultrasound, Doppler ultrasound and computerized tomography. Inclusion criteria were 1) age> 18 years, 2) receiving no treatment for cirrhosis, 3) using propranolol. The exclusion criteria were 1) structural heart disease, 2) heart ventricular pacing, 3) electrolyte abnormalities, 4) using the drugs affecting QTi.

### Data collection

Baseline characteristics of the participants including: age, gender, and Model for End-Stage Liver Disease (MELD) score were obtained by a date gathering form. Both groups underwent Twelve-lead ECG, and 2D echo, TDI (vivid E9 echo machine, GE, USA).

#### QTd measurement

Twelve-lead ECG was obtained in both cirrhotic and healthy groups. The QT interval was measured on the basis of millisecond from the beginning of QRS complex into the end of T wave, based. Then, the corrected QT (QTc) was calculated based on heart rate using the MDCalc^©^ 2005-2018 online calculator, using Bazett’s formula. Lastly, considering the difference between minimum and maximum QTc values in the ECG the QTd was measured in millisecond.

#### MCd measurement

2D echo and tissue Doppler study by a vivid E9 echo machine was done. Three views of four-chamber, apical three-chamber and two-chamber with color coded TDI study was obtained and saved. Then in an offline mode 12 segments of basal and mid parts of myocardium were evaluated and myocardial contractility duration in each segments was measured. Myocardial contractile duration was calculated from the time of start of QRS wave to the end of S wave (the last positive wave that crosses zero line). Subsequently the minimum and maximum of it in 12 segments were determined and the difference between them was considered as MCd.

### Outcomes

The main outcome of this study was to compare the MCd in two groups of cirrhosis patients and healthy individuals. The second outcome was to compare myocardial contraction duration between two groups among 12 segments of LV. Lastly, we evaluated QTd between the two groups.

### Statistical analysis

All statistical analyses were performed with the Statistical Package for Social Sciences (SPSS) version 17.0 (SPSS Inc., Chicago, IL, USA). Data will be presented as mean ± standard deviation (SD). Chi-square tests, or Fisher exact test were used to compare the proportions in two groups. Also, the mean of myocardial contraction duration, MCd, and QTd were compared with independent-sample *t* test between two groups. Pearson correlation test was used for investigating any correlation between MELD score and MCd as well as QTd. *P* value<0:05 was considered statistically significant.

## Results

### Baseline characteristics

Sixty individuals (40 male/20 female) with a mean age of 40.1 ± 7.1 years were studied in two groups of cirrhotic patients (n = 30) and healthy individuals (n=30). Both groups were statistically similar in terms of age (Cirrhotic vs. Healthy: 41.03±6.64 vs. 39.16±7.57: *P*=0.31) and gender (Cirrhotic vs. Healthy [male/female]: 19/11 vs. 21/9: *P*=0.39). MELD score of the cirrhotic patients was 16.4±5.2 (range 8-28). Seven patients (23.4%) had a MELD score ≥20, 21 (70.0%) 9<score<20, and 2 patients (6.6%) score ≤9.

### Myocardial contraction duration

The longest myocardial contraction duration was observed in the base of lateral segment (326.3 ± 35.9 ms) in cirrhotic group, while it was in the middle of posterior segment (329 ± 48.3 ms) in healthy individuals. No significant differences were reported for myocardial contraction duration between two groups regarding 12 LV segments (Table 1).

**Table 1:** Myocardial contraction duration between two study group among 12 segments of LV

**Myocardial contraction duration (ms), mean ± SD**		**Cirrhotic patients (n=30)**	**Healthy individuals (n=30)**	***P*** ** value**
Septum	Base	322±37.17	320.3±42.7	0.31
	Middle	321.6±38.4	318±39.4	0.71
Anterior	Base	317±35.8	322±34.7	0.56
	Middle	318.6±35.7	323.6±38.1	0.49
Anteroseptal	Base	314.6±40.3	326.3±44.9	0.29
	Middle	312.3±36.9	325.6±44.5	0.21
Lateral	Base	326.3±35.9	322±43.1	0.67
	Middle	320.6±37.6	309.3±57.7	0.37
Inferior	Base	309.9±65.7	324±38.3	0.31
	Middle	315.3±35.9	323.6±38	0.38
Posterior	Base	320.3±38.7	328.3±46.6	0.47
	Middle	317.6±36.8	329±48.3	0.31

### MCd and QTd

**Figure 1:**
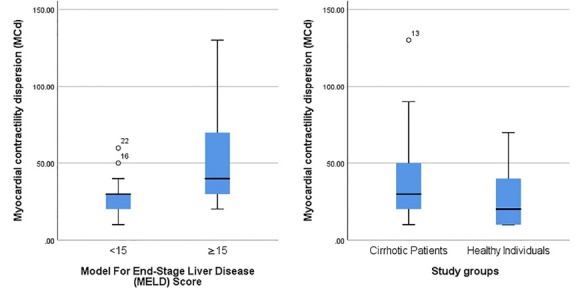
Comparison of myocardial contractile disepresion.

MCd of cirrhotic patients was significantly higher than healthy individuals (41.0±26.8 versus 27.6±18.1 millisecond; *P* = 0.028) (Figure 1). Moreover, the cirrhotic patients had significantly higher QTd compared to the healthy individuals (37.0±22.1 versus 25.3±8.9 ms; *P* = 0.010). MCd was reported to be higher than QTd in both cirrhotic patients (*P* = 0.06) and healthy individual groups (*P* = 0.28).

### MELD score

Cirrhotic patients with MELD score<15 had a lower MCd in comparison to score ≥15 (MCd: 29.2±13.8 versus 50.0±31.1, *P* = 0.034, Figure 1). The QTd was also reported lower in patients with MELD score<15 (26.1±9.6 versus 45.3±25.5 ms, *P* = 0.016). Pearson correlation analysis revealed that MELD score of cirrhotic patients was correlated with MCd (r = 0.554, *P* = 0.002) and QTd (r = 0.554, *P* = 0.002).

## Discussion

The primary aim of this study was to investigate the MCd and QTd changes in cirrhotic patients in comparison with healthy individuals. Moreover, we wanted to evaluate MCd and QTd relationship with cirrhosis severity. Our results demonstrated that both MCd and QTd were significantly higher in cirrhotic patients. Also, a significant association between MCd and QTd as well as MELD score, as an approved index for cirrhosis severity was founded out for the first time. Although, to best of our knowledge using the TDI for evaluation the MCd, was not investigated so far; but the measurement of QTc and QTd as indicators of ventricular repolarization prolongation and dispersion have been widely used during the last two decades.^4,7,8^

The electrical abnormalities commonly described include changes in the QTi and QTc, associated with the evolutionary stages of the cirrhosis.^11,12^ It has been suggested that the QTi may represent a prognostic marker of autonomic dysfunction and survival in CCM.^13^ Although the mechanism of these cardiac abnormalities is not fully understood yet, it is postulated that these alterations are because of molecular alterations of conductive system and myocardial muscles of the heart.^14,15^ Another important factor in determining the electrocardiographic abnormalities of cirrhotic patients is to eliminate the other causes of these disturbances, including ischemia, valvular heart disease, restrictive or congenital heart disease, conduction abnormalities, arterial hypertension, lung disease, using anti-arrhythmic drugs, and active consumption of alcoholic beverages.^2,9^ Up to now, QTc interval prolongation has been documented in the evolutionary stages of the cirrhosis; however, we tried to introduce some other factors such as QTd and MCd. Zahmatkeshan et al in a study which was conducted on pediatric patients with end-stage liver disease demonstrated that the patients with end-stage liver disease had significantly longer QTc compared to the controls.^16^ Moreover, the results of Genovesi et al demonstrated that QTc was progressively prolonged from Child A to Child C patients.^12^ Nevertheless, we did not assess QTc association with the presence or severity of cirrhosis, and we could not compare the results of other studies with ours.

An increased QTd has been reported as an arrhythmogenic risk factor in various clinical groups, although the evidences about its association with cirrhosis was not enough so far. So, our results should be considered as another piece of the puzzle, which made a great contribution to for the association between increased QTd and cirrhosis. Our results were concordant with the results from all studies, however they had only estimated QTd with ECG. Also, identification of the end of the T-wave is difficult and measurements of dispersion by ECG have modest reproducibility.^17^ Another study evaluating pediatric with cirrhosis showed that the patients with end-stage liver disease had significantly longer QT dispersion compared to the controls.^16^ Moreover, Zamirian et al compared QTd before and after liver transplant; their results showed that QTd had significantly decreased after liver transplant.^8^

In our study, we evaluated myocardial contraction duration by TDI echocardiography instead of QTc resulted ECG. The rational with TDI was to quantify precisely myocardial velocities and to perform regional measurements. According to our knowledge, our study was the first one which attempt to clarify the myocardial contraction duration between healthy and cirrhosis groups. Although, myocardial contraction duration was somehow higher in cirrhotic patient at the septum and lateral parts of LV, the remaining parts were higher in healthy individuals; however, no significant difference was reported regarding contraction duration in 12 LV segments of basal and middle parts. We believe that the lower severity of the disease, which showed by MELD score less than 20, in our cirrhotic patients’ groups was one of the major probable reasons for no difference of contraction duration between healthy and cirrhotic patients.

Some previous studies have indicated an association between Child-Pugh or MELD score and the QTc interval, but the majority of studies have not shown any correlation.^18,19^ In fact, considering the low number of high quality studies, the association between the severity of the liver disease and cardiac impairment is not entirely clear. Our results demonstrated that QTd and MCd of the cirrhotic patients was positively correlated with MELD, and QTd and MCd of the patients with MELD >15 were significantly more than those in the patients with MELD<15. MELD was originally used to determine the priority of organ transplant recipients.^20^ It was later modified to estimate the severity of cirrhosis in pre-transplant patients; increased MELD score was closely associated with increased mortality. So, we concluded that the values of QTd and MCd increased with severity of cirrhosis. Undoubtedly, our finding is difficult to interpret and could probably result from the small number of patients; hence, we need to increase the number of evaluations. This was in contrast with the results from Zahmatkeshan et al^16^ reported the lack of associations between MELD score as well as Child- Pugh score with the QTc interval and QTd. Although the different study population, which was adult in ours and pediatric in their studies, might be the possible cause of the results heterogeneity.

There were several limitations in our study. The first major limitation was about the sample size of our study. We tried to increase the number of studied patients, more than the expected sample size, but the number of patients with our considered inclusion criteria were limited. Moreover, we could not assess the prognostic importance of MCd and QTd; since we could not follow-up our patients, considering their outcomes. Furthermore, TDI is an angle dependent which makes it sensitive to malalignment between principle direction of myocardial shortening and Doppler beam. This limitation can also explain reasons for no difference of MCD between healthy and cirrhotic patients in this study. Finally, in our study we could not exclude cirrhotic patients which receive beta blockers because these drugs are necessary for them and discontinuation of them would raise ethical problems. Consequently, the effect of these drugs on QT interval cannot be ruled out. We recommend further studies, of course with larger sample size, and longer follow-up, to reassess the association between TDI findings and severity of cirrhosis, beside its outcome.

## Conclusion

Based on the results of this study, cirrhosis was associated with increased MCd, assessed by TDI. Also, MCd and QTd were associated with a higher MELD score, as a marker of cirrhosis severity. According to the results, it seems that MCd and QTd might be the possible predictor of ventricular arrhythmia in patients with cirrhosis; however, further cohort studies are needed to prove the prognostic value of these variables in the outcome of cirrhotic patients.

## Competing interests

Authors declare no conflict of interest in this study.

## Ethical approval

The study protocol was approved by the institutional review board (IRB) and the Ethics Committee of Shiraz University of Medical Sciences. The content of the project was explained to all patients before entering the study, and they signed the consent form. Privacy and dignity of patients were also considered in the project. Information on patients was inserted into the statistical analysis software as encoded, and was published as overall results.

## Acknowledgements

The present study has been adopted from Specialty thesis written in Cardiology by Dr. Hamed Mirzaee at Shiraz University of Medical Sciences (Code: 9750). This study was conducted with the financial and spiritual supports of the Deputy of Research at Shiraz University of Medical Sciences.
